# GO annotation in InterPro: why stability does not indicate accuracy in a sea of changing annotations

**DOI:** 10.1093/database/baw027

**Published:** 2016-03-19

**Authors:** Amaia Sangrador-Vegas, Alex L. Mitchell, Hsin-Yu Chang, Siew-Yit Yong, Robert D. Finn

**Affiliations:** ^1^European Molecular Biology Laboratory, European Bioinformatics Institute (EMBL-EBI), Wellcome Trust Genome Campus, Hinxton, Cambridge, CB10 1SD, UK

## Abstract

The removal of annotation from biological databases is often perceived as an indicator of erroneous annotation. As a corollary, annotation stability is considered to be a measure of reliability. However, diverse data-driven events can affect the stability of annotations in both primary protein sequence databases and the protein family databases that are built upon the sequence databases and used to help annotate them. Here, we describe some of these events and their consequences for the InterPro database, and demonstrate that annotation removal or reassignment is not always linked to incorrect annotation by the curator.

**Database URL:** http://www.ebi.ac.uk/interpro

## Introduction

**Figure 1.**
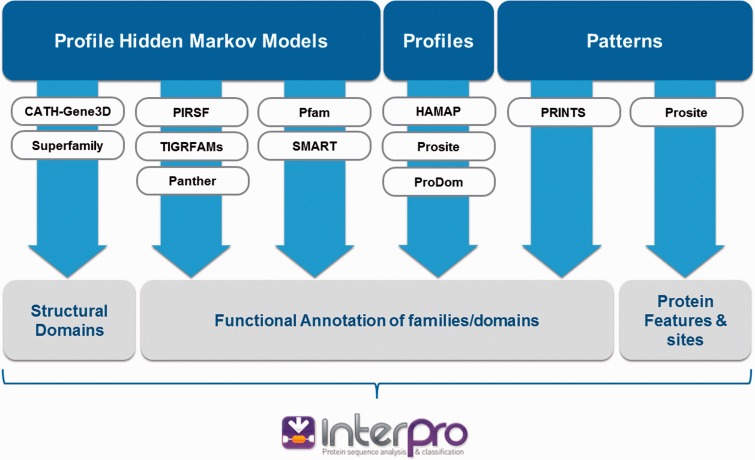
InterPro integrates signatures from 11 member databases. Each source database has its own distinct biological focus (structural domains, functional families/domains, protein sites) and uses different methods to create protein signatures (hidden Markov models, profiles or patterns).

InterPro (1) integrates 11 protein family databases (CATH-Gene3D (2), HAMAP (3), PANTHER (4), Pfam (5), PRINTS (6), ProDom (7), PROSITE Patterns (8), PROSITE Profiles (8), SMART (9), SUPERFAMILY (10) and TIGRFAMs (11)) that each provide models (either hidden Markov models, profiles, position-specific score matrices or regular expressions; referred to collectively as ‘signatures’) to help classify proteins. Each source database has its own distinct biological focus and*/*or method of signature production (Figure 1). They offer complementary levels of protein classification, from broad-level (e.g. a protein is a member of a superfamily) to more fine-grained assignments (e.g. a protein is a member of a particular family or possesses a specific type of domain).

**Figure 2.**
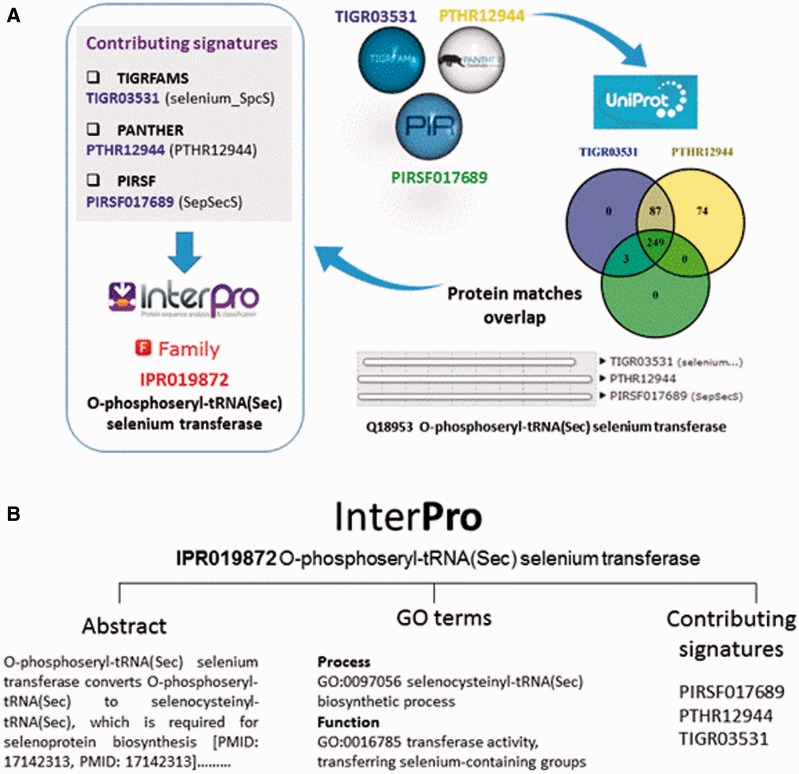
(**A**) Signatures are integrated in the same InterPro entry when they describe the same protein family or domain. In this example, three signatures from member databases TIGRFAM, PANTHER and PIRSF describing a family of O-phosphoseryl-tRNA selenium transferases are integrated together into InterPro entry IPR019872. Entries can have one or several contributing signatures. Integrating signatures contributes to reduce redundancy and to rationalize the wealth of information from the different member databases. (**B**) InterPro entries include an abstract and GO annotation, when possible.

The InterPro database does not generate signatures itself, but groups one or more related member database signatures into InterPro entries to provide a single, comprehensive resource for understanding protein families, domains and functional sites. Once a member database signature is categorized by InterPro, that database signature is considered ‘integrated’ (Figure 2A). The assignment of signatures and the relationship between entries is determined by curators, who inspect the matches between the signatures and Swiss-Prot, the manually annotated subsection of UniProtKB (12) (or matches to TrEMBL, the unreviewed section of UniProtKB, if no Swiss-Prot match is found). InterPro match data feed back into UniProtKB and play a key role in the automated analysis and annotation of sequence data held in TrEMBL.

**Figure 3.**
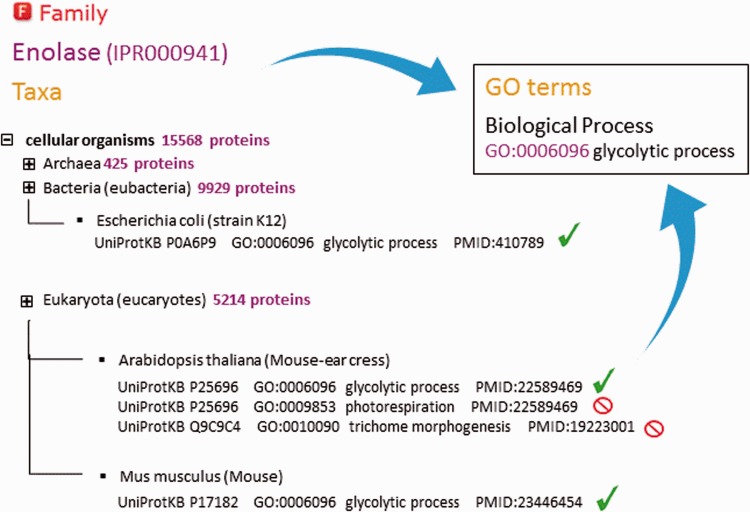
Assignment of ‘biological process’ GO terms to InterPro entry IPR000941 (enolase). InterPro curators decide which terms can be applied by analysing the spectrum of proteins from different organisms matched by that entry. Existing annotation for individual proteins that is supported by experimental evidence is considered. Certain GO terms are only applicable to a restricted taxonomic group (e.g. ‘photorespiration’ cannot be applied to animals and ‘trichome morphogenesis’ can only be applied to plants). GO terms assigned to an entry should be applicable to all the proteins matched by that entry.

As part of the added information provided by InterPro, curators annotate each database entry with literature-referenced abstracts and additional functional annotations, including Gene Ontology (GO) (13) terms where possible (Figure 2B). The GO represents a controlled vocabulary that can be used to describe genes and gene products in a consistent and structured fashion. The GO terms are structured within a directed acyclic graph (DAG), and each term has defined relationships to one or more other terms in the same domain (i.e. biological process, molecular function or cellular location), and sometimes across domains. InterPro’s GO annotation is based both on the experimental evidence available for characterized proteins and the taxonomic range of proteins matched by a particular InterPro entry (e.g. plant- specific GO terms cannot be added to an InterPro entry that matches proteins from organisms other than plants (Figure 3)). Once a GO term is applied to an InterPro entry, it is automatically propagated to all the UniProtKB proteins matched by that entry (the InterPro2GO pipeline). This process enables the transfer of GO-based functional information from a relatively few experimentally characterized sequences to a set of evolutionarily related (as determined by the matches to the signature) but as yet uncharacterized sequences.

Most GO annotations in the UniProtKB database are assigned electronically, and there is an interest in estimating the reliability of these terms. One measure used to assess reliability has been annotation stability (14). The idea here is that the removal of a GO annotation indicates that the original annotation was erroneous and should be considered a negative reliability indicator. However (as with most active biological databases), InterPro data are in constant flux, with changes to signatures, integrations and underlying protein sequences. Furthermore, GO terms are not static and are constantly being added to and improved (15). As a result, InterPro annotations are never completely stable. Here, we investigate some of the different data-driven events that can affect the stability of electronic annotations provided by the InterPro database.

## Drivers of InterPro annotation changes

### Case 1: updates to member databases

When a member database produces a new release, signatures within the database may be changed. In the most extreme cases, pre-existing signatures may be deleted altogether. For example, updates to PANTHER (which is InterPro’s largest member database in terms of number of signatures) have involved the introduction of new phylogenetic tree reconstruction algorithms and subfamily boundary refinements, which have resulted in the removal of signatures from the database (16). PANTHER version 10.0, the latest PANTHER version at the time of writing, included 22 new genomes in their phylogenetic trees, expanding from 82 to 104 genomes; 250 families were merged into other larger families, and refinements to subfamily identification were applied (17). Consequently, the update of PANTHER version 9.0 to 10.0 involved substantial changes to the database, resulting in deletion of 23 836 signatures (representing PANTHER families or subfamilies) and the addition of 59 006 new signatures.

Many InterPro entries (75% approximately) consist of just one signature. In those cases, if the signature is deleted after a member database update and there is no equivalent to replace it (i.e. no signature from any member database matching the same set of proteins), the signature-less InterPro entry becomes ‘empty’ and is deleted. This does not mean the signature was wrong. Updates to member databases aim to improve protein coverage and classification, but changes can affect signatures that were originally well defined. For example, entry IPR028413 (suppressor of cytokine signalling) consisted of PTHR10385 before the PANTHER 10.0 update. This signature was deleted in PANTHER version 10.0, and replaced with a signature that represents a broader family, PTHR24369. This caused the removal of orphaned entry IPR028413 from InterPro 54.0 and the loss of annotations for the GO term originally assigned to this entry, ‘GO:0035556 intracellular signal transduction’. Overall, as a result of the PANTHER 10.0 update, 332 InterPro entries were left signature-less and were removed, resulting in the loss of 522 InterPro2GO annotations, affecting 76 430 UniProtKB proteins, with an overall loss of 178 926 GO annotations to proteins.

In less severe cases, existing signatures may be altered as part of a member database release. For example, a profile hidden Markov model may be rebuilt or the threshold used to prevent false-positive matches to a signature may be changed in order to refine the sequence set matched (either to match a tighter functionally related group of proteins, or to recognize more distant homologues). InterPro entries that undergo significant protein match changes are investigated following member database updates. For example, following the PANTHER 10.0 update, in addition to reviewing the signature-less entries (which accounted for 332 entries that were lost and 700 that were assigned another signature), 675 entries had to be manually reviewed because they had significant protein match changes. Entry annotations and GO terms were modified by curators where required, who may select more or less specific terms from the GO hierarchy with which to reannotate the InterPro entry, depending on the nature of the protein match changes.

### Case 2: new and changed sequences in the UniProtKB database

Changes in InterPro annotation can also arise as a result of updates to the UniProtKB sequence database. UniProt updates are released monthly, where new sequences enter the database and others are deleted or updated. The addition or removal of sequences from UniProtKB can result in gains or losses of matches to existing InterPro entries. These changes are manually reviewed by InterPro curators after each UniProt update and InterPro annotations are updated where necessary. Curators focus on changes affecting the Swiss-Prot (reviewed) protein set matched by an InterPro entry, changes to the taxonomic range of the proteins matched, or substantial changes in the total number of proteins matching an entry. For example, InterPro entry IPR030545 represents the WD-repeat-containing protein 62 (WDR62) family. WDR62 is required for cerebral cortical development in vertebrates (18). The contributing signature (PTHR22847:SF434) matched 71 proteins from Metazoa in UniProt release 2014_11. However, the term ‘GO:0022008, neurogenesis’ was removed from the InterPro entry when UniProt 2015_01 was released, as the signature was then found to match 18 additional fungal uncharacterized proteins that had been added to UniProtKB as part of that release. These non-vertebrate matches may indicate that the signature represents a taxonomically broader family than previously thought, or may represent false-positive matches that need to be eliminated by increasing the stringency of the signature threshold. Ultimately, the underlying issue is that new protein sequences have become available and now match this signature. Consequently, the neurogenesis term had to be removed to prevent erroneous annotations from being created, despite the fact that it was perfectly valid at the time of assignment.

### Case 3: increased scientific knowledge and understanding

UniProtKB updates functional information about proteins in the database as new data becomes available in the scientific literature. Following a UniProt update, newly characterized proteins can be a source of additional annotation for InterPro entries, and sometimes new information about protein function requires existing GO annotations to be amended. For example, InterPro entry IPR012864 (Cysteine oxygenase/2-aminoethanethiol dioxygenase), based on Pfam signature PF07847, matches proteins from both animals and plants. When this entry was created in 2011, the animal homologues were annotated as cysteamine dioxygenases, while the plant homologues were uncharacterized. As a result, the term ‘GO:0047800 cysteamine dioxygenase activity’ was assigned to this InterPro entry. However, as part of UniProtKB release 2015_05, the plant sequences were reannotated as cysteine oxidases based on experiments described in a recent publication (19). Despite the similarity between the animal and plant homologues (∼46% amino acid similarity based on pairwise Basic Local Alignment Search Tool (BLAST) comparisons between representative sequences from each group of homologues), they therefore appear to possess different catalytic functions. Consequently, the corresponding InterPro entry annotation was updated and the more general GO term ‘GO:0016702 oxidoreductase activity, acting on single donors with incorporation of molecular oxygen, incorporation of two atoms of oxygen’ (a less functionally specific ancestor term of ‘GO:0047800’ in the GO hierarchy) was assigned to replace ‘GO:0047800’. This change resulted in updated GO annotations for almost 1000 UniProtKB proteins, following InterPro update 54.0.

**Figure 4.**
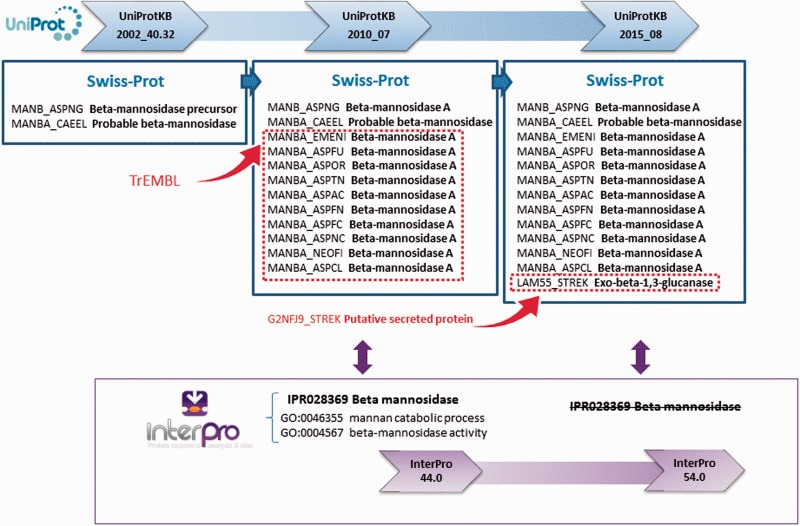
UniProt updates reflect the current knowledge about proteins. As new information emerges from scientific publications, sequences previously uncharacterized can be assigned a function. InterPro entries change according to current knowledge, and in some cases they have to be deleted, as in this example for entry IPR028369. After a previously putative secreted protein was characterized as an exo-beta-1,3-glucanase, IPR028369 was deemed non-specific in terms of the proteins it was matching and was removed from the database. The GO annotation associated with this entry was consequently lost.

Changes in the annotation assigned to individual proteins can also cause an InterPro entry and/or its GO terms to be removed. This is part of the natural dynamic of protein signature databases, where signatures can have a ‘life cycle’ and may need to be replaced as new sequences and/or scientific information becomes available. For example, the InterPro entry IPR028369 (beta-mannosidase), based on PANTHER signature PTHR10066:SF12, was created in September 2013 as part of InterPro release 44.0. In UniProt release 2015_08, the annotation for G2NFJ9 (a putative secreted protein in TrEMBL) was manually updated and this protein was moved into Swiss-Prot, after a new publication determined the sequence to be an exo-beta-1,3-glucanase (20) (Figure 4). As a result of this additional information, it became apparent that IPR028369 was not only matching beta-mannosidases (members of the glycosyl hydrolase 2 family), but also some members of the glycosyl hydrolase 55 family (exo-beta-1,3-glucanases). As the boundaries for this family were not well defined—it matched exo-beta-1,3-glucanase G2NFJ9, but not other glycosyl hydrolase 55 members—the InterPro entry was removed from the database and the contributing signature reverted to being unintegrated. As a result, the GO terms ‘GO:0046355 mannan catabolic process’ and ‘GO:0004567 beta-mannosidase activity’ were lost for 2981 matching proteins. This exemplifies how annotation and signatures have to be revisited and sometimes modified to reflect the current biological knowledge.

### Case 4: modifications to the GO

The GO itself is also regularly updated by the GO editorial team, based on their own work and suggestions from the research community (21). This can result in the creation of new, more specific GO terms for describing protein function, and these new annotations are routinely incorporated into InterPro. An example of this is illustrated by IPR012536 (cytomegalovirus US glycoprotein), based on Pfam signature PF08001, which matches a group of unique short cytoplasmic glycoproteins that can integrate into host endoplasmic reticulum membrane and inhibit host immune response (22). The InterPro entry was created in 2005, and the term ‘GO:0030176 integral component of endoplasmic reticulum membrane’ was assigned. In 2011, a more precise term, ‘GO:0044386 integral to host endoplasmic reticulum membrane’, was created by the GO editorial team. This newer term subsequently substituted the previous GO term, as part of an update to the annotation of IPR012536. As a result, the term GO:0030176 was lost for 200 UniProtKB proteins, but they each received the newer, more descriptive term instead.

## Discussion

Outlined above are a series of cases where the GO annotations provided by the InterPro database were updated or removed, but not necessarily due to original annotation inaccuracies or unreliability. Rather, the changes reflect a continuous effort by curators to provide the highest quality and most up-to-date annotations. InterPro is not the only database to face such issues; UniProt who, similar to InterPro, provides GO annotations to proteins have also described similar misconceptions regarding their annotations (15).

In InterPro’s case there are at least four sources of information that are in constant flux: (i) the protein sequences in the database, (ii) the state-of-the art scientific knowledge about their function, (iii) the protein family signatures provided to InterPro by its member databases and (iv) the GO itself. As illustrated, changes to any one of these information sources can result in annotation changes of varying scale, and the cumulative effect of changes to several sources at the same time can be vast. Users should be aware that annotations and entries can change with each release of InterPro. A typical InterPro release involves changes to at least two of these sources—an update to the latest version of UniProtKB and an update of at least one member database—and may therefore result in annotation changes for (many) thousands of proteins.

Further complicating matters for curators is deciding what GO annotation should be applied to an InterPro entry; it requires information to be sourced and scrutinized, relating to groups of proteins that may have diverse functions and/or be drawn from wide taxonomic groupings. Since curation is a manual activity, performed by human curators, the annotations selected can be subjective. To help maintain annotation consistency, all new InterPro entries are independently checked by a second curator before integration into the database. InterPro curators also regularly review existing entries and check the UniProtKB sequence matches to identify discrepancies that conflict with entry annotation. They also systematically scan the scientific literature and review novel publications, adapting InterPro annotation as new information emerges and the function of certain proteins becomes clearer. Often, changes in InterPro entry-assigned GO terms reflect a simple step either up or down the GO term DAG, in light of new data.

Undeniably, sometimes annotation is removed or changed because it was not correct in the first place, but this represents a small fraction of the number of terms deleted or changed in each release. InterPro curators receive regular feedback through the GO-annotation tracking system, which is used by members of the GO community to highlight misannotations and to suggest annotation improvements. Such procedures and systems are vitally important for maintaining annotation quality, especially in the case of InterPro, where assigned GO terms are propagated to many millions of proteins.

**Figure 5.**
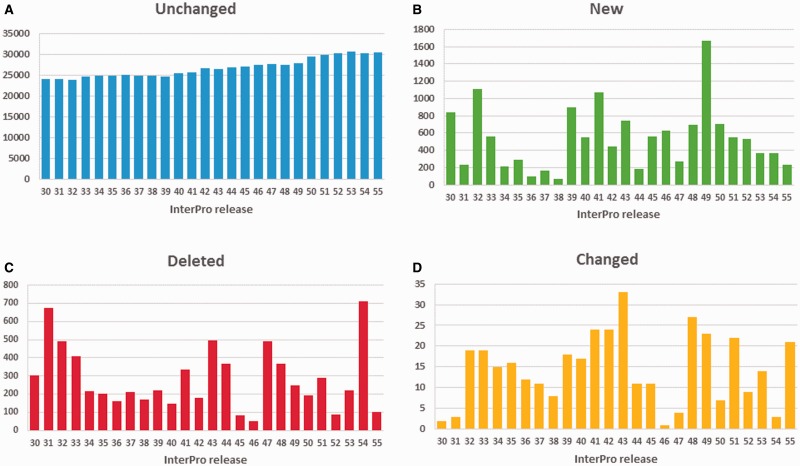
Number of GO annotations to InterPro entries: (**A**) unchanged, (**B**) added, (**C**) deleted and (**D**) changed (step up or down the GO term DAG) from InterPro release 30.0 (December 2010) till release 55.0 (December 2015). Releases 31.0, 44.0, 47.0 and 54.0 included updates to PANTHER, the largest member database in terms of number of signatures.

While annotation changes can affect a large number of proteins, the vast majority of InterPro GO annotations are stable, as illustrated in Figure 5A. Spikes in new annotations (Figure 5B), deleted (Figure 5C) and changed (where a GO term is replaced by either a more or less specific term from the DAG, Figure 5D), tend to correspond to large-scale member database changes, such as major PANTHER updates (as can be seen at and following InterPro releases 31.0, 47.0 and 54.0). Overall, the number of GO annotations in the database has been steadily increasing with consecutive InterPro releases. To date, InterPro release 55.0 included 30 706 GO terms mapped to InterPro entries, with 5370 distinct GO terms.

## Conclusion

InterPro integrates the signatures from its member databases into a single resource to create a powerful protein analysis tool. Furthermore, it also generates functional annotation over and above the contributions from its member databases, including GO terms. The InterPro2GO pipeline provides >108 million GO term associations to UniProtKB at release 2016_01, representing one of the main sources of GO annotation. InterPro GO annotations are largely stable, but the dynamic nature of the underlying data sources means that changes are necessary. As discussed in this paper, annotation instability, at least in terms of InterPro-derived annotation, should not be considered to be a signifier of unreliability. Instead, the annotation changes described underline the vital importance of continued curation, where the annotation of database entries should never be considered completely resolved. Only through ongoing vigilance and updating of entries by curators can databases hope to keep pace with ever-changing biological knowledge.

## Funding

This work was supported by the Biotechnology and Biological Sciences Research Council (BBSRC) [BB/L024136/1]; the Wellcome Trust [108433/Z/15/Z]; European Molecular Biology Laboratory (EMBL) core funds. Funding for open access charge: EMBL core funds.

*Conflict of interest*. None declared.
